# Probing the Structure, Stability and Hydrogen Adsorption of Lithium Functionalized Isoreticular MOF-5 (Fe, Cu, Co, Ni and Zn) by Density Functional Theory

**DOI:** 10.3390/ijms10041601

**Published:** 2009-04-14

**Authors:** Natarajan Sathiyamoorthy Venkataramanan, Ryoji Sahara, Hiroshi Mizuseki, Yoshiyuki Kawazoe

**Affiliations:** Institute for Materials Research(IMR), 2-1-1, Katahira, Aoba-Ku, Sendai, Japan 980 8577; E-Mails: sahara@imr.edu (R.S.); Mizuseki@imr.edu (H.M.); kawazoe@imr.edu (Y.K.)

**Keywords:** Density functional Theory (DFT), Metal-organic frameworks (MOF’s), Hydrogen storage: Li-functionalization

## Abstract

Li adsorption on isoreticular MOFs with metal Fe, Cu, Co, Ni and Zn was studied using density function theory. Li functionalization shows a considerable structural change associated with a volume change in isoreticular MOF-5 except for the Zn metal center. Hydrogen binding energies on Li functionalized MOFs are seen to be in the range of 0.2 eV, which is the desired value for an ideal reversible storage system. This study has clearly shown that Li doping is possible only in Zn-based MOF-5, which would be better candidate to reversibly store hydrogen.

## 1. Introduction

Nowadays, worldwide interest is focused on using a clean burning substitute such as hydrogen in place of fossil fuels, due to both economic and environmental reasons [1,2]. However, hydrogen storage is one of the most important challenges impeding its practical application. Physisorption on large surface area materials is one significant approach for storing hydrogen. Some metal-organic frameworks (MOFs) show promising results for hydrogen adsorption, but the storage pressure and temperature conditions make their use economically unfavorable [3–5].

In order to improve the storage conditions, it is important to design new MOF materials having not only tailored pore dimensions and large void volumes, but also incorporating strong adsorption sites, like for instance, adsorbing alkali metals on the surface [6–10]. In addition, researchers have proposed that metal centers present in the MOFs play an important role in the gas adsorption property [11–13]. Recently, Li doping was performed to enhance the storage capacity of MOFs both by experimental and theoretical means and to provide the desired binding enthalpies in the range of 0.2 – 0.3 eV for hydrogen [14–18].

**Scheme 1.**
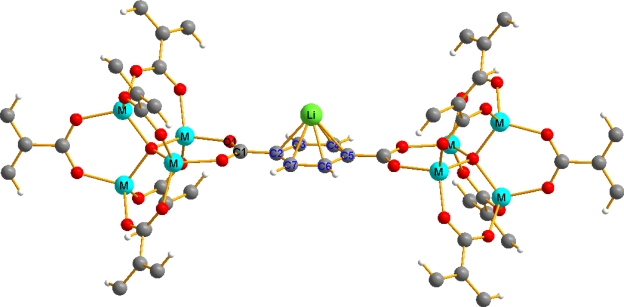
Primitive unit cell of isoreticular MOF-5 (M = Fe, Co, Ni, Cu, Zn). Colors: blue (Metal center), gray (C atoms), white (H atoms), green (Li atom), red (oxygen). The structure is formed by OM_4_ tetrahedra at the corners linked by benzene dicarboxylic (BDC) groups.

In the report, by means of first-principles calculations, we have studied the binding energies for hydrogen molecules on the Li functionalized Zn-MOF-5. Furthermore, we have made a study on Li-functionalized isoreticular M-MOF-5’s characterized by the presence of different metal centers (Scheme 1) to find the stability and structure of MOF’s after doping the M-MOF-5 unit (M = Fe, Co, Ni Cu and Zn) with Li atoms. The paper is organized as follows. In the first part, the effect of Li doping on the MOF-5 unit is explored by doping Li atom. In the second part we have studied the binding of hydrogen molecules on the Li functionalized Zn-MOFs. In the third part we have studied the structure and stability of lithium functionalized isoreticular MOF-5 and our conclusions are given in Section 4.

## 2. Results and Discussion

The structure of the primitive cell representing the unit cell was fully optimized without any geometrical constraints. In Table 1 we compare the present computational result of the MOF-5 structure with experimental data, which are in good agreement. This provides confidence in our computational method.

### 2.1. Stability of the Li doping on MOF’s

To examine the effect of Li doping, we first investigated the adsorption energy of doping Li atom on the benzene unit of Zn-MOF-5. A summary of Li adsorption energy on Zn-MOF-5 can be seen in Table 1. The energy gain in attaching Li to a MOF-5 unit is called the adsorption energy (ΔAE) and is defined as follows:

(1) ΔAE=E(Li−MOF)−E(Li)−E(MOF)

where *E*(Li-MOF), *E*(Li) and *E*(MOF) are the total energies of the unit cell containing the adsorbed Li atoms and the energy of the MOF-5 unit respectively. A positive value of *ΔAE* would indicate an energy gain in attaching the Li to the MOF-5 surface, and the negative value is energy lost during the process. From our calculations it is evident that doping Li atom was found to be exothermic in nature. In addition a significant change in the bond length of benzene unit was observed, which was attributed to the charge transfer from the Li cation to the MOF-5 unit. Furthermore, up on Li functionalization only a slight change in structure and bond parameters is found to occur near OZn_4_ tetrahedra site. The calculated Li atom - benzene adsorption energy was lower than the reported values of 1.6 eV by Dixon and co-workers [19]. Such a decrease in expected as there exists effective conjugation between OZn_4_ tetrahedra unit and linker Benzene dicarboxylic (BDC) unit. Bader charge analysis on the system revealed, that Li atom carries a +0.9e charge over it. It is important to specify here that Rao and Jena had shown that a positive charge atom can bind a large amount of hydrogen that by a neutral atom by charge polarization mechanism [20].

### 2.2. Hydrogen adsorption on Li-functionalized MOF’s

**Figure 1.**
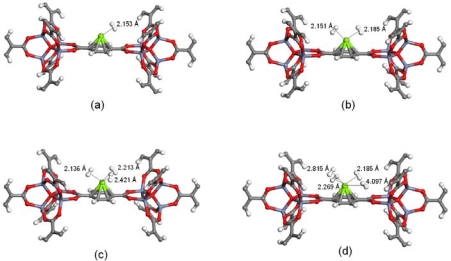
Optimized geometries of adsorbed hydrogen molecules on Li functionalized Zn-MOF-5 with one (a), two (b), three (c) and four (d) hydrogen molecules. Colors grey (C atoms), white (H atoms, red (O atoms) green (Li atoms) pale gray (Zn atoms).

To understand the effect of hydrogen adsorption on the Li-functionalized MOF’s we have optimized the Li-functionalized Zn-MOF-5 with hydrogen molecules near to the Li atom. Upon structural relaxation only slight change in structure and bond parameters was found to occur on the organic linker and near OZn_4_ tetrahedra. The hydrogen interaction or the binding energy per hydrogen molecule (*ΔE_b_*) can be defined as:

(2) $$\Delta E_{b}=[E_{T}(\text{Li}-\text{MOF})+E_{T}({\text{H}}_{2})-E_{T}(\text{Li}-\text{MOF}+{\text{H}}_{2})]/{\text{nH}}_{2}$$

where *E_T_*(Li-MOF+H_2_) and *E_T_*(Li-MOF) refer to the total energy of the Li- functionalized Zn-MOF with and without hydrogen molecule respectively, while the *E_T_*(H_2_) refers to the total energy of the free hydrogen molecule and “n” is the number of hydrogen molecules. In the current work we have added one to four hydrogen molecules near the Li center. The calculated structural parameters and the binding energies are provided in Table 2, while the optimized structures are provided in Figure 1.

For the first H_2_, the interaction energy is 0.213 eV, with an intermolecular distance of 2.153 Ǻ. This binding energy per atom was close to the value reported for systems that can be used for an ideal reversible hydrogen storage system [22].The orientation of hydrogen is in T-shape, with a H-H bond distance of 0.760 Å, which corresponds to a very small change compared to the 0.750 Å bond length in a free hydrogen molecule. This indicates that the Li cation holds the H_2_ molecules by a charge – quadruple and charge – induced dipole interaction [23]. When the second hydrogen is introduced, the Li – H_2_ distance increases to 2.124 Å, while the binding energy per H_2_ molecule gets reduced to 0.209 eV. To know the number of hydrogen molecules a Li cation can hold, we doped the third and fourth H_2_ near the Li cation. With the introduction of third H_2_ the Li – H_2_ distance increases along with a decreases in the interaction energy value. A noticeable feature is the H – H distance which decreases with the increase in the number of H_2_. When the fourth hydrogen is introduced near the Li cation, three H_2_ are place near the Li atom and the other H_2_ molecule is moved away to a nonbonding distance of 4.036 Å. Thus each Li cation can hold up to 3 hydrogen molecules in a quasi molecular form. Furthermore, each BDC unit can adsorb one Li atom on each face and can maximize its storage to 6 hydrogen molecules per BDC unit. Further the existence of several metal sites will increase the storage capacity of the Li-functionalized MOF’s.

### 2.3. Effect of metal center on Li-functionalized MOF’s

To know the possibility of extending the Li doping to other IRMOF-5, we studied the adsorption of Li cation on IRMOF-5. We have selected metals (M= Fe, Co, Ni, Cu and Zn) from the same column of the periodic table by replacing Zn centers. Structural relaxation without Li atom shows only a slight change in volume to occur up on the change in metal sites. However, up on doping with Li atom a considerable change in shape and structure was observed for other materials except Zn. Table 3 contains structural information for the linker and the benzene-Li mean distance. One can observe that the linker unit benzene remains practically unchanged in all the studied compounds.

In Table 4. we have collected the M-O1, C1-O1, C1-C2 bond lengths, O1-M-O2 bond angles and the adsorption energy of Li for the optimized structure of M-MOF-5 system. From the table it is evident that a significant structural change is observed around the OM_4_ tetrahedra unit and linker BDC unit. The metal-metal distance deviation is shorter in the case of Zn, and very high reduction in distance is observed for iron system. Further, calculated adsorption energy for these compounds don’t show any regular trend with the metals. These results suggest that adsorption energy of Li not only depend on the metal centers but are greatly influenced by the structural change and the volume of the system that changes upon Li doping.

## 3. Computational Methods

The calculations were performed using density functional theory (DFT) in the generalized gradient approximation (GGA) with the exchange-correlation function proposed by Perdew–Wang (PW91) [24] as implemented in the VASP plane wave. To represent the atomic cores, we use the projector augmented wave pseudopotentials. Ionic positions and cell parameters were relaxed with respect to minimum forces and stress by using conjugated-gradient algorithms. All atoms were fully relaxed with the energy converged to less than 10^−5^ eV. Electronic wave functions are represented by plane waves up to 400 eV. Calculations were preformed on a 106-atom, primitive cell of dimension 26 Å X 13 Å X 13 Å which imposes periodic boundary conditions. As we are dealing with a large system, the Γ-point was used for sampling the Brillouin zone.

## 4. Conclusions

In summary, we have performed a DFT study to determine the stability of Li atom binding on Zn-MOF. The adsorption energy calculated shows that Li atom adsorption is exothermic in MOF system. Hydrogen adsorption on Li functionalized Zn-MOF-5 occurs with only a small structural change. Doping Li enhances the binding energy of hydrogen molecules to the desired value. Each Li was found to hold up to three hydrogen molecules in a near molecular form. Introduction of Li on isoreticular MOFs with metals Fe, Co, Ni, Cu shows a considerable structural change, to occurs near the OM_4_ tetrahedral site, leading to the reduction in the volume of the materials. The adsorption energy and stability of Li doping on these materials not only depend on the metal centers by are greatly influenced by the structural change and the volume of the system that changes after Li doping.

## Figures and Tables

**Table 1.** Adsorption energy (AE, eV) and selected bond parameters (Å) for Li atom on Zn-MOF-5 unit.

System	*ΔAE* eV	C2–C3^a^	C3–C4^a^	C4–C5^a^	C5–C6^a^	C6–C7^a^	C7–C2^a^	C_M_–Li^b^
Li–Zn-MOF	−1.56	1.428	1.380	1.428	1.428	1.380	1.428	2.229
Zn-MOF^c^		1.399 (1.388)	1.384 (1.375)	1.399 (1.392)	1.399 (1.397)	1.384 (1.379)	1.399 (1.392)	

^a^For numbering see Scheme 1.

^b^Mean Li–C distance

^c^Values in parenthesis are experimental values from ref. [21].

**Table 2.** Selected bond parameters (Å) and binding energy per hydrogen molecule (eV) for the adsorption of hydrogen on Li functionalized Zn-MOF.

No. of H_2_	Avg. Benzene – Li (Å)	Li – H_2_ (Å)	Avg. H – H (Å)	*ΔE*_*b*_ (eV)
0	2.206	-	0.750	-
1	2.223	2.096	0.760	0.213
2	2.241	2.124	0.759	0.209
3	2.257	2.315	0.755	0.196
4	2.252	2.379 (4.036)	0.755 (0.751)	0.163

**Table 3.** Calculated structural parameters (Å) in the organic linker of Li cation doped M-MOF-5 (M = Fe, Co, Ni, Cu, Zn).

System	C2–C3	C3–C4	C4–C5	C5–C6	C6–C7	C7–C2	C_M_–Li^a^
Fe-MOF-5	1.425	1.369	1.425	1.425	1.396	1.425	2.274
Co-MOF-5	1.417	1.407	1.417	1.417	1.407	1.417	2.292
Ni-MOF-5	1.410	1.391	1.410	1.410	1.391	1.410	2.280
Cu-MOF-5	1.407	1.396	1.408	1.407	1.394	1.404	2.285
Zn-MOF-5	1.436	1.377	1.437	1.437	1.377	1.436	2.209

^a^Mean benzene – Li distance

**Table 4.** Selected bond lengths (Å) bond angles (deg) and adsorption energy of Li on M-MOF-5 (M = Fe, Co, Ni, Cu, Zn).

System	M–O1	C1–O1	C1–C2	O1–C–O2	M1–M2	ΔAE eV
Fe-MOF-5	1.915	1.289	1.479	123.6	2.881	0.621
Co-MOF-5	2.010	1.276	1.527	125.4	2.399	0.548
Ni-MOF-5	1.949	1.270	1.481	127.1	2.864	0.776
Cu-MOF-5	2.009	1.265	1.501	128.2	2.939	3.14
Zn-MOF-5	1.956	1.264	1.491	129.5	3.128	1.15
Zn-MOF-5^a^	1.937 (1.911)	1.274 (1.300)	1.479	129.3 (125.0)	3.128 (3.160)	-

^a^Calculated and experimental values (in parenthesis) for Zn-MOF-5 without Li doping. Experimental values are from ref. [20].
